# High-risk infrastructure projects pose imminent threats to forests in Indonesian Borneo

**DOI:** 10.1038/s41598-018-36594-8

**Published:** 2019-01-15

**Authors:** Mohammed Alamgir, Mason J. Campbell, Sean Sloan, Ali Suhardiman, Jatna Supriatna, William F. Laurance

**Affiliations:** 1 Centre for Tropical Environmental and Sustainability Science, and College of Science and Engineering, James Cook University, Cairns, Queensland 4878 Australia; 2 Laboratory of Forest Inventory and Planning, Faculty of Forestry, University of Mulawarman, Samarinda, 75123 East Kalimantan Indonesia; 3 Research Center for Climate Change, and Department of Biology, Faculty of Math and Sciences, University of Indonesia, Depok, 16424 Jakarta Indonesia

## Abstract

Indonesian Borneo (Kalimantan) sustains ~37 million hectares of native tropical forest. Numerous large-scale infrastructure projects aimed at promoting land-development activities are planned or ongoing in the region. However, little is known of the potential impacts of this new infrastructure on Bornean forests or biodiversity. We found that planned and ongoing road and rail-line developments will have many detrimental ecological impacts, including fragmenting large expanses of intact forest. Assuming conservatively that new road and rail projects will influence only a 1 km buffer on either side, landscape connectivity across the region will decline sharply (from 89% to 55%) if all imminently planned projects proceed. This will have particularly large impacts on wide-ranging, rare species such as rhinoceros, orangutans, and elephants. Planned developments will impact 42 protected areas, undermining Indonesian efforts to achieve key targets under the Convention on Biological Diversity. New infrastructure will accelerate expansion in intact or frontier regions of legal and illegal logging and land colonization as well as illicit mining and wildlife poaching. The net environmental, social, financial, and economic risks of several imminent projects—such as parallel border roads in West, East, and North Kalimantan, new Trans-Kalimantan road developments in Central Kalimantan and North Kalimantan, and freeways and rail lines in East Kalimantan—could markedly outstrip their overall benefits. Such projects should be reconsidered in light of rigorous cost-benefit frameworks.

## Introduction

Infrastructure expansion is occurring at a dramatic rate across the globe. Paved roads have increased by ~12 million km worldwide since 2000, with an additional ~25 million km projected by mid-century^1,2^. Countries with extensive tropical forest areas are becoming the epicentres of this infrastructure expansion^3–5^, threatening many of the world’s biologically richest ecosystems^6–11^. Globally, 110 million hectares of tropical forest were lost between 2000 and 2012, frequently in the aftermath of infrastructure expansion^12^.

**Figure 1:**
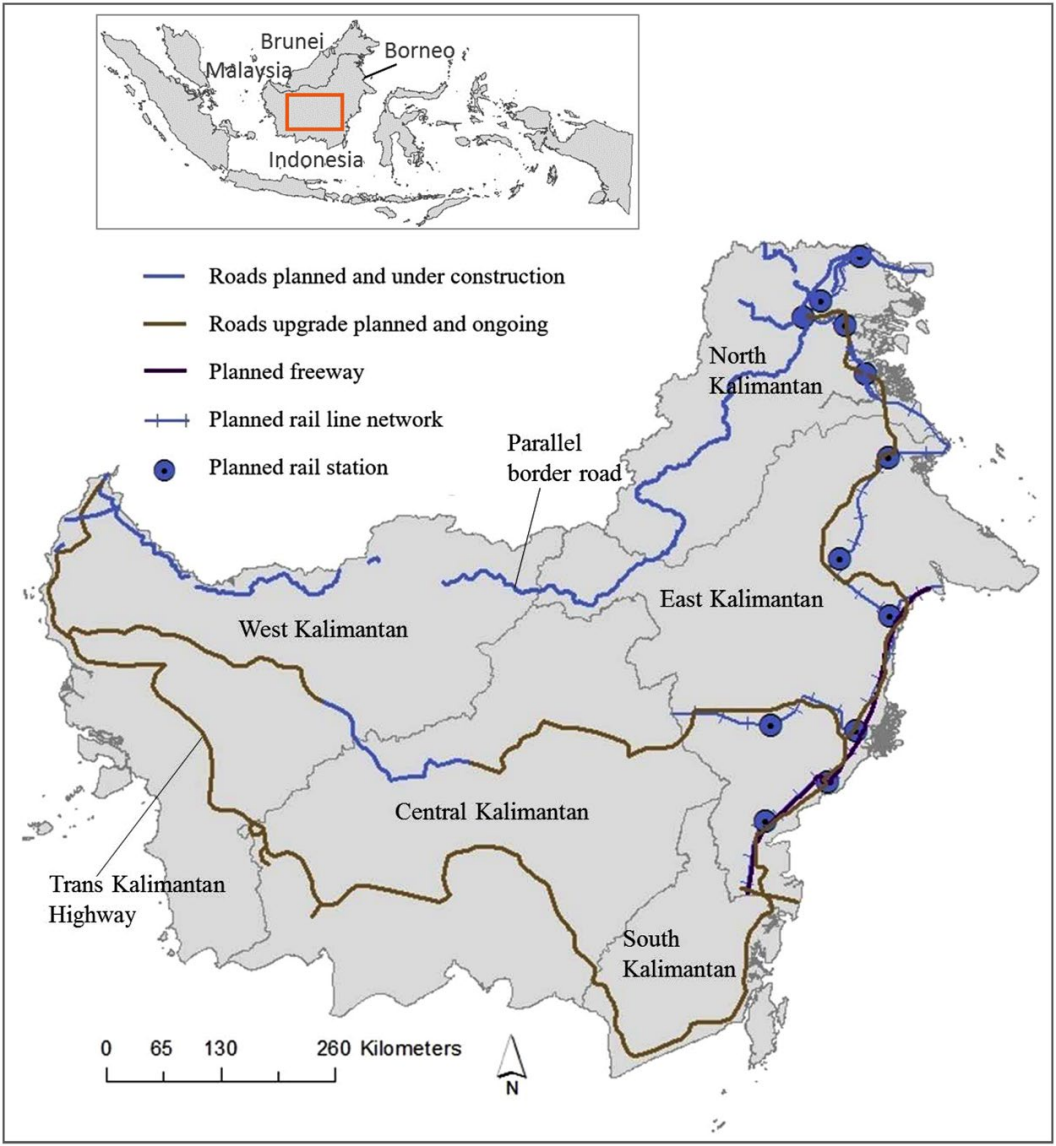
Major planned and ongoing infrastructure-expansion networks in Indonesian Borneo examined in the current study. Parallel border roads were extracted from Indonesian Government Infrastructure Information maps for each five provinces of Kalimantan^31–35^. Trans-Kalimantan Highway data were digitized from Potter^38^ and the Indonesian Master Plan^26^. Additional roads in North and West Kalimantan were digitized from ADB^71^. Spatial data on planned freeway, new roads, rail lines, and rail stations in East and North Kalimantan were obtained from the Department of Forestry, Mulawarman University, East Kalimantan. The maps were created using Esri ArcMap 10.4.1 (https://www.arcgis.com).

Borneo (Fig. 1) sustains the largest intact forest area in Southeast Asia. These forests harbour a global biodiversity hotspot, exceptionally high species endemism, and large carbon stocks. They also provide important ecosystem services for local and indigenous communities. The ~37 million hectares of tropical forest in Indonesian Borneo (Kalimantan)^13^ comprises diverse ecosystems including montane forests, lowland mixed-dipterocarp forests, and peat-swamp forests^14–18^. These forests have been undergoing clearance and degradation at varying but generally high rates^19–21^ since the onset of industrial-scale extractive industries half a century ago^22^. Since 1973, ~31% of all remaining forests have been lost^22^, with primary (old-growth) forests being the worst affected, declining by ~14.4 million hectares^23^. Key drivers include infrastructure expansion, logging, oil palm and wood-pulp plantations, mining, and wildfires associated with major droughts^9,22,24^.

Indonesia, the most extensively forested Southeast Asian nation, experienced the world’s highest annual rate of forest loss (2.2 million hectares) in 2011–2012, overtaking Brazil^12^. Over the same period, its road network increased by ~42%, with legally mapped roads expanding by ~151,000 km in length^25^. Many roads have markedly increased nearby forest loss and degradation, particularly those built through and adjacent to wilderness areas^4,9,24^. Such trends are slated to increase further under the Indonesian Master Plan for the Acceleration and Expansion of Economic Development (2011–2025)^26^, which aims to sharply expand road infrastructure by 2025 via the development of six major economic corridors across the Indonesian archipelago^26,27^. One of these, the Kalimantan Economic Corridor, will sharply expand transportation infrastructure across Kalimantan^26^.

Among the infrastructure projects planned or underway in Kalimantan are an upgrade of 3,316 km Trans-Kalimantan Highway in southern Kalimantan^26,28,29^, 1,920 km of new roads (parallel border roads) in northern Kalimantan flanking the Malaysian-Indonesian border^29–35^, and additional highways and expressways in Central, South, East, and North Kalimantan^26,29,36–38^ (Fig. 1). The primary goal of this new infrastructure is to increase commercial connectivity and primary industries, particularly coal mining, oil palm, and industrial logging^26,39^. However, the major impacts of this spate of new infrastructure and extractive industries on native forest, biodiversity and relevant attributes—forest fragmentation, landscape connectivity and current forests pattern— in Kalimantan have not been quantified.

For instance, Kalimantan’s forests are host to one of the two remaining habitat on the planet where orangutan, rhinoceros and elephant co-exist^40^. It also harbours several globally endangered species (e.g., Bornean orangutans (*Pongo pygmaeus*), pygmy elephants (*Elephas maximus borneensis*))^41^ and iconic species (e.g., bearded pigs (*Sus barbatus*), sun bears (*Helarctos malayanus*)) that require a large home range to maintain viable population^42^. Consequently, if planned and ongoing infrastructure expansion fragments forests and reduces landscape connectivity, it will likely seriously impact the key species and landscape ecological dynamics in Indonesian Borneo.

Here we evaluate how planned and ongoing infrastructure development in Indonesian Borneo could impact the spatial pattern, connectivity, protected areas, and overall environmental integrity of the region’s native forests. This preliminary analysis underscores a number of potentially serious threats to ecosystems in the region.

## Results

### Infrastructure expansion and forest spatial pattern

**Figure 2:**
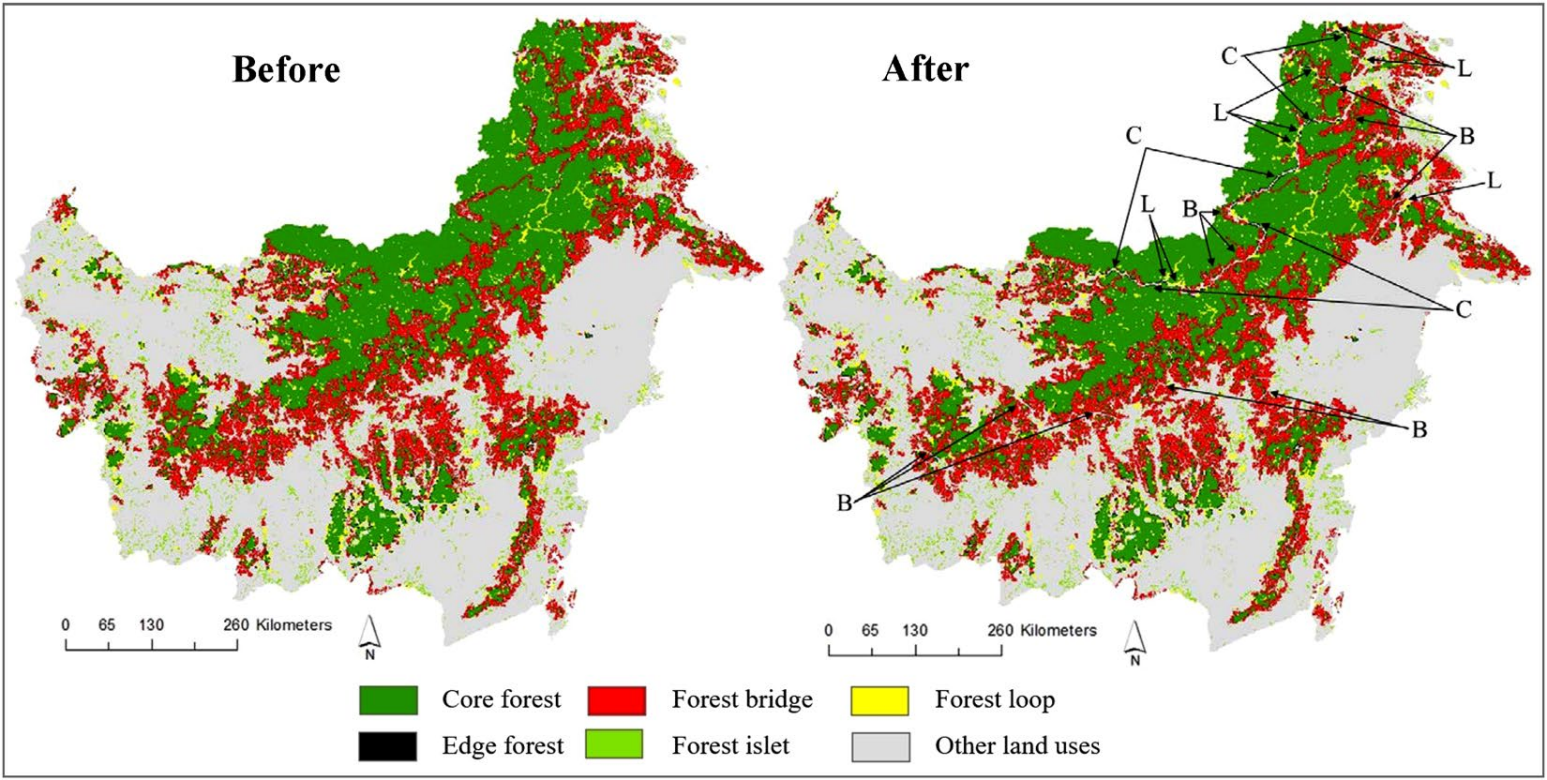
Planned and ongoing infrastructure expansion and the projected changes in forest spatial pattern in Indonesian Borneo. The left and right panel show spatial pattern of forests before and after infrastructure expansion, respectively. “Core forest” indicates relatively intact forest patches ≥900 m from the nearest forest edge; a “forest loop” is a connecting pathway between two sections of a core forest; a “forest bridge” connects different core forests; a “forest islet” is an isolated forest patch too small to contain core area; and “edge forest” is <900 m from the nearest forest edge. The letters in the right panel indicate where infrastructure expansion would dissect core forests (C), bridge forest (B), and loop forest (L). The maps were created using Guidos Toolbox 2.6 version 4 (http://forest.jrc.ec.europa.eu/download/software/guidos/).

Planned and ongoing road development will substantially alter the current spatial pattern of forests in Indonesian Borneo. Infrastructure expansion will create an additional ~97,000 ha of forest-edge habitat (forest <900 m from the nearest forest edge) — a 17% increase over current levels. Conversely, ~237,000 ha of core forests (forest ≥900 m from an edge) will be transformed into other, non-core forest categories, primarily small forest patches and edge forests, due to increased forest fragmentation or clearing associated with infrastructure development (Fig. 2). Furthermore, ~392,000 ha of existing ‘bridge’ forest corridors connecting different core-forest areas (Fig. 2) will be impacted, potentially ecologically disconnecting large expanses of core forest from one another. Finally, ~90,000 ha of existing ‘loop’ forests — those that connect different sections of a core-forest patch (Fig. 2) — will be created because of increased fragmentation. All these findings clearly indicate that planned and ongoing road development in Indonesian Borneo will substantially alter the current spatial pattern of forests, leading to large-scale degradation and fragmentation of currently intact forest and a marked decline of forest connectivity (Fig. 2).

### Infrastructure expansion and landscape connectivity

**Figure 3:**
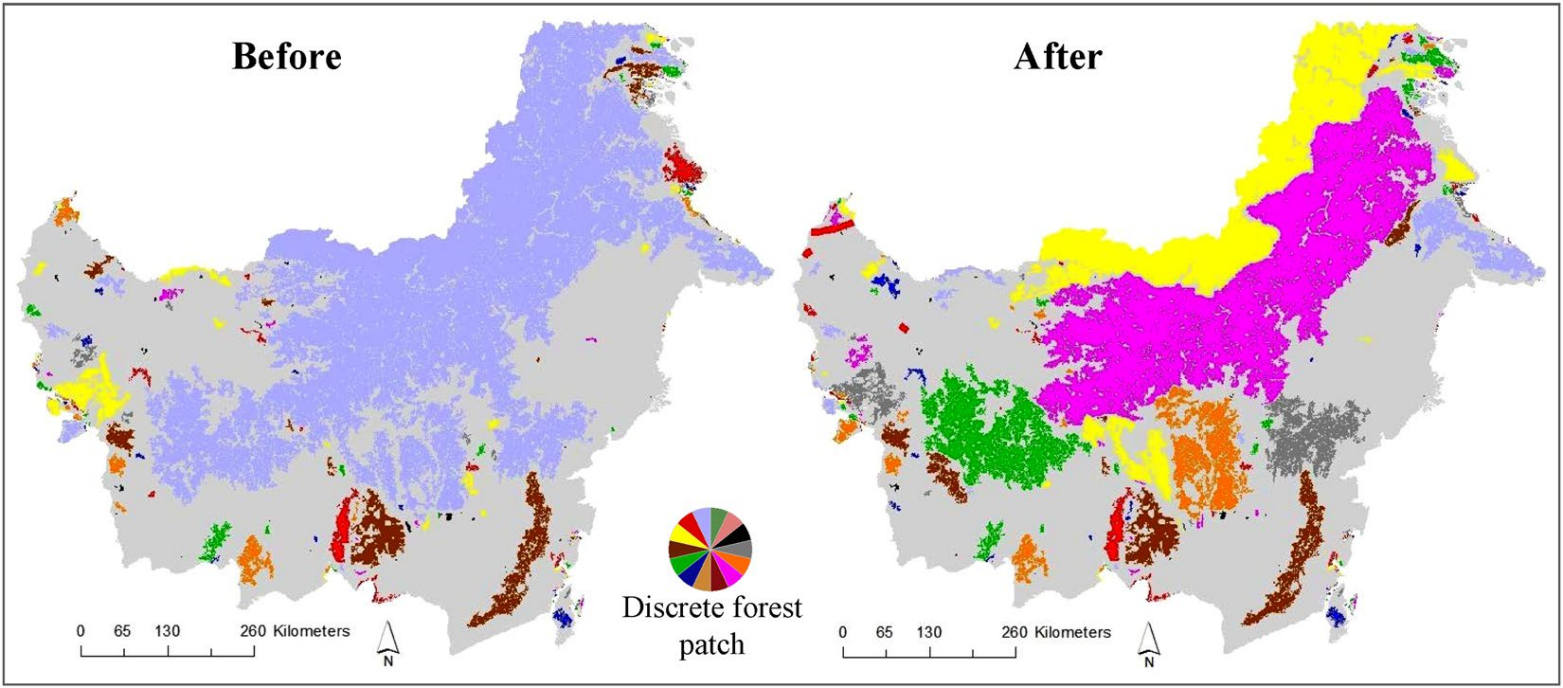
Planned and ongoing infrastructure expansion and the projected decline in landscape connectivity in Indonesian Borneo. The left and right panel show landscape connectivity before and after infrastructure expansion, respectively. Different colours in a map indicate an isolated forest patch. The maps were created using Guidos Toolbox 2.6 version 4 (http://forest.jrc.ec.europa.eu/download/software/guidos/).

Planned and ongoing road development in Indonesian Borneo will significantly reduce the connectivity of forests at regional scales. Our analysis shows that the Equivalent Connected Area (ECA) — a measure of the percentage of habitat accessible by wildlife based on the degree of network connectivity^43^— is currently 89% for forested areas of Indonesian Borneo. If the planned and ongoing infrastructure expansion were to occur, this index would be reduced to 55% (Fig. 3), meaning that more than one-third (34%) of the accessible habitat in the region would be isolated from the remaining accessible habitat. This would create a number of additional isolated habitat patches across the Kalimantan region (Fig. 3, right panel). This process of patch isolation would predominantly occur via the bisection of larger habitat patches and destruction of numerous forest linkages that currently connect core-forest areas (Fig. 2).

### Infrastructure expansion and protected areas

**Figure 4:**
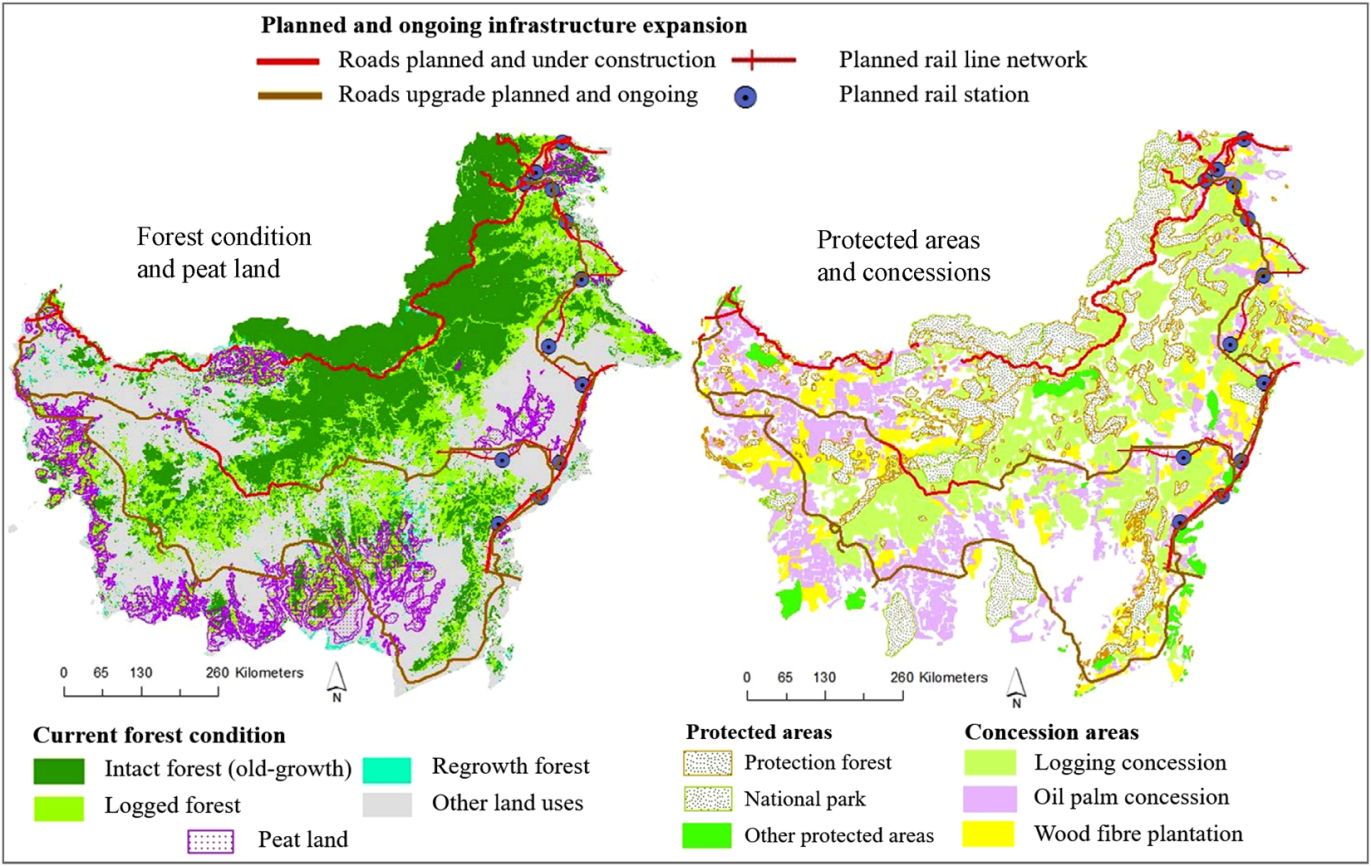
Planned and ongoing infrastructure-expansion routes with respect to current forest condition, peatland, protected areas, and concessions in Indonesian Borneo. In the figure, other protected areas include nature reserves, wildlife reserves, sanctuary reserves, citizen forestry parks, and nature recreation parks that each comprise less than 5% of all protected areas. The maps were created using Esri ArcMap 10.4.1 (https://www.arcgis.com).

The planned and ongoing road expansion would have a major impact on existing protected areas in Indonesian Borneo. Using high-resolution data (30 m-pixel size) on protected areas, we found that planned and ongoing construction of new road and rail lines would intersect a total of 25 existing protected areas that are currently free from major road incursion (Fig. 4). Notably, a planned parallel border-road project in north Kalimantan will intersect a relatively intact protected area — Kayan Mentarang National Park — that is among the largest remaining protected areas in Indonesian Borneo (Fig. 4). Planned and ongoing projects would reduce the internal ecological connectivity of the protected areas and also the extensive trans-boundary connectivity between Indonesian Borneo and Malaysian Borneo (Figs 1 and 4). In addition to new infrastructure project, planned upgrades of existing roads will further impact 17 protected areas, particularly in southern Borneo (Fig. 4). This process would increase disturbances from earth- and road-works, increase erosion and stream sedimentation, and increase barrier effects^44^ to wildlife by widening existing clearings inside protected areas.

### Infrastructure expansion and logging, oil palm, and wood fibre industries

The planned and ongoing road expansion in Kalimantan is likely to facilitate logging, oil palm, and wood-fibre industries in intact forest frontiers. We found that most of the planned and ongoing road developments are situated either inside or along the margins of primary or selectively logged forests adjacent to logging, oil palm, and wood-fibre concessions (Fig. 4). This pattern suggests that much road expansion will be at the expense of native forest. We also observed that many ongoing and planned road developments are located along the margins of concessions adjacent to remaining primary or selectively logged forests (Fig. 4).

### Infrastructure expansion and environmental consequences

**Figure 5:**
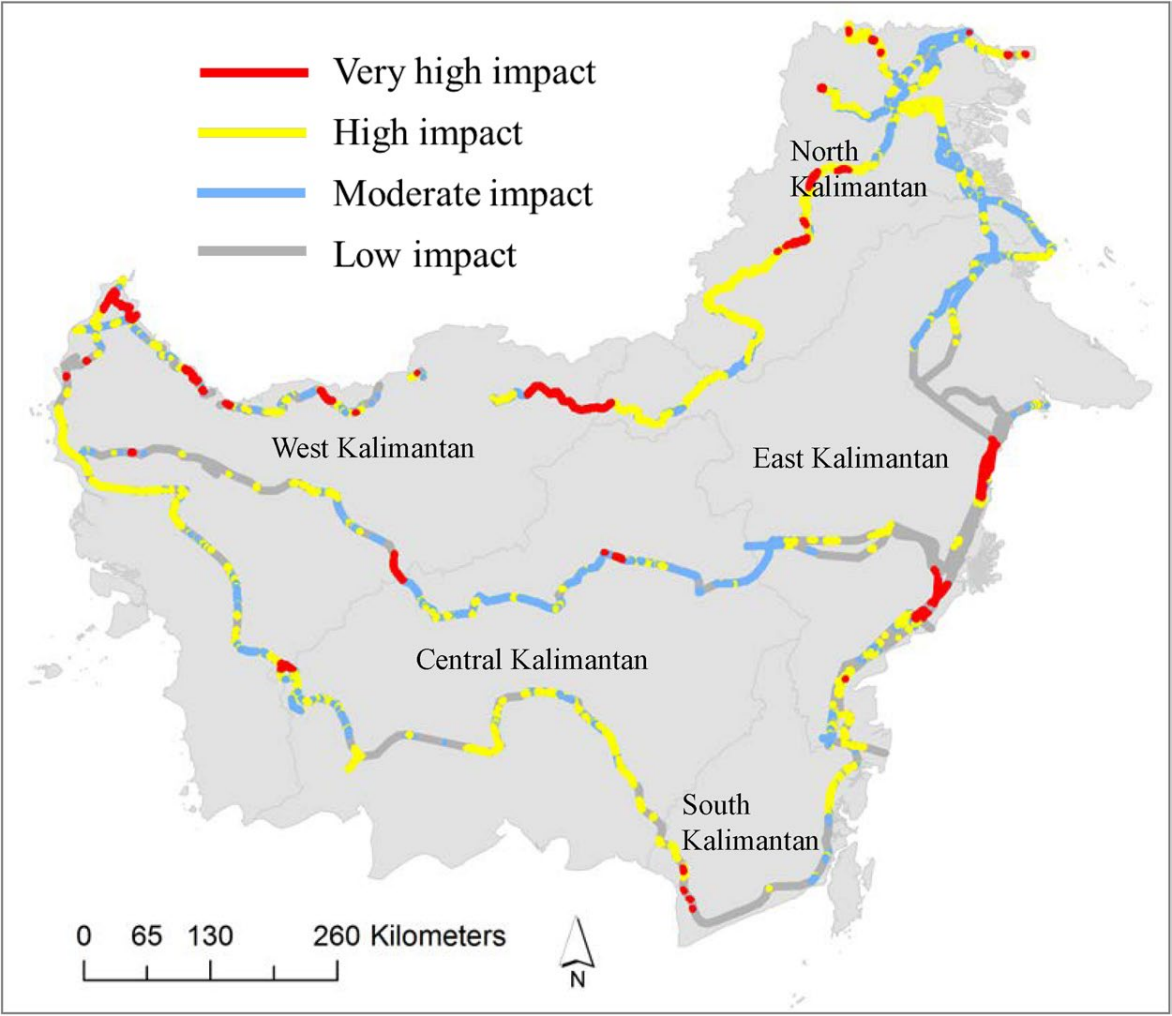
Categorization of planned and ongoing road and rail lines by their potential environmental impacts in Indonesian Borneo. The maps were created using Esri ArcMap 10.4.1 (https://www.arcgis.com).

Different segments of the planned and ongoing road and rail lines in Kalimantan have varying environmental risks. We classified planned and ongoing road and rail lines construction and upgrades into different categories, considering their overall potential implications for regional environmental integrity. Our analyses classified 634 km of new and upgraded road and rail lines in Kalimantan as *very high impact* i.e. these roads and railine segments will bisect current protected areas (Fig. 5). Furthermore, 1,472 km are classified as *high impact*, as these segments will cut through primary forest or peat land. Additionally, 1,242 km segments are classified as *moderate impact*, indicating that these segments will cut through selectively logged forests or regrowth forests (Fig. 5). This categorization indicates that a substantial part of the planned construction and upgrade locations in Kalimantan coincides with current protected areas, peat land, intact forests, and selectively logged and regrowth forests.

## Discussion

The planned and ongoing expansion of infrastructure in Kalimantan will significantly degrade large expanses of native forest. It will sharply increase edge forests and forest fragmentation while reducing core forest (Fig. 2). Our findings are consistent with several previous analyses (for example, refs^5,11^) showing that road proliferation in tropical landscapes dramatically increases forest fragmentation and abrupt forest edges. The projected changes in spatial pattern will occur because a number of ongoing and planned infrastructure projects, such as parallel border road projects in West, East, and North Kalimantan, will cut through large core-forest areas. Such large-scale fragmentation will inevitably diminish key environmental and ecological values, such as disturbance-sensitive biodiversity, carbon storage, and hydrological functioning^45–48^. The fragmented forests would be more vulnerable to subsequent forest conversion via legal and illegal means, mirroring trends observed in the Amazon^49^, Sumatra^4^, and Malaysian Borneo^50^.

Indices of landscape connectivity measure the proportion of suitable habitat in a landscape that is accessible by wildlife populations. Our findings—including a marked loss of current forest linkages (Fig. 2), the creation of a number of isolated forest patches, and a 34% decline in overall landscape connectivity (Fig. 3)—indicate that planned projects would have major impacts on forest connectivity. The consequences of this severe connectivity loss may be dire for the unique biodiversity of Borneo, particularly for several globally endangered animals that have large home ranges, such as Bornean orangutans (*Pongo pygmaeus*) and pygmy elephants (*Elephas maximus borneensis*), as well as iconic species such as bearded pigs (*Sus barbatus*) and sun bears (*Helarctos malayanus*) that will travel hundreds of kilometers in response to mast fruiting^41,42,51^. The critically endangered Sumatran rhinoceros (*Dicerorhinus sumatrensis*) may also persist in tiny numbers in Kalimantan. Planned projects could pose severe threats to the viability of these rare and space-demanding species, in part by massively reducing forest connectivity between Malaysian and Indonesian Borneo, which have long existed as a single forest block.

Many wildlife species — including many species actively poached and traded illegally — would become more vulnerable to poachers and hunters from increased road access and forest fragmentation. Similar consequences were reported from African tropical forests where forest elephants (*Loxodonta cyclotis*) declined by more than 60% between 2002 and 2011, largely due to the increased access for poachers and commercial hunters from forest-road expansion^52–54^. In Peninsular Malaysia, ~90% of snares and poaching camps in a studied forested area were found within 5 km of a paved road^55^. Similarly, Sumatran orangutans (*Pongo abelii*) and Tapanuli orangutans (*Pongo tapanuliensis*) in Sumatra are both critically endangered by infrastructure proliferation in their remaining habitat^4,10,56^. Beyond these iconic species, infrastructure would also negatively affect forest-interior small mammals, arboreal species, and birds^44,57–59^, among others.

Our analyses suggest that planned and ongoing infrastructure expansion in Kalimantan will be highly disruptive to protected areas, by diminishing their connectivity to nearby forests and increasing encroachment and poaching. If all planned infrastructure expansion proceeds, 42 protected areas will be impacted to varying degrees (Fig. 4). This process will undermine the Indonesian commitment to achieve the Aichi Target 11 of the Convention on Biological Diversity that aims to ensure that by 2020 at least 17% of the total terrestrial area will be represented by well-connected protected area systems^60^. Both at the regional (Kalimantan) and national scale, Indonesia is currently well behind schedule to achieve this target and immediate actions to maintain or improve protected-area connectivity are essential^61,62^. Road construction facilitates deforestation and degradation of protection forest, and is one of the main causes of declining effectiveness of protected areas across Indonesia^63^ and the world^64^.

If major infrastructure projects in Kalimantan proceed as planned, the majority of remaining old-growth forests (unlogged primary forests) and peatlands in Kalimantan may become accessible, threatening their near-term degradation (Fig. 4). This would parallel current and historical trends of deforestation in Borneo^21–23^ and elsewhere in Indonesia^21^. The current Indonesia Master Plan (2011–2025) as presently framed will lead to sharp increases in forest loss and disruption and reductions in their associated environmental services, such as the high levels of carbon storage in upland and peatland forests, which would have global implications for climate change^65,66^.

If completed as planned, the overall environmental risks posed by infrastructure expansion in Indonesian Borneo would be extremely high. Our categorization of over 3,300 km of planned and ongoing road and rail lines as *very high impact*, *high impact*, and *moderate impact*, underscores the high environmental risks involved in current development schemes. We assert that new road and rail-line segments categorized as *very high impact* should not proceed on environmental grounds. Upgrading of road and rail line segments categorized as *high impact* and *moderate impact* should be implemented only with a focus on limiting their environmental impacts via stringent mitigation and offsets measures, the establishment of new protected areas along road routes^8^, and improved law enforcement following road development^67,68^. New road and rail-line segments categorized as *moderate impact* should be implemented with a clear focus on limiting their impacts, following proactive land-use zoning, and ensuring appropriate mitigation and offset measures^69,70^ and stringent monitoring and law enforcement. The extremely high environmental cost of road segments could provide a rationale for curtailing or cancelling the projects. The parallel border road projects in West Kalimantan, East Kalimantan and North Kalimantan provinces, and the development of Trans-Kalimantan Highway segments in Central Kalimantan, South Kalimantan and East Kalimantan provinces (Fig. 5), are likely to contribute most substantially to environmental degradation.

The goal of current infrastructure expansion in Kalimantan is to enhance economic development and integration^26^ by 2025, with an explicit assumption that infrastructure expansion is necessary for the economic growth of the region. Infrastructure expansion in Kalimantan is clearly needed but must be strategically located and designed, considering unique environmental and other attributes of the region^69,70^. Otherwise, it can open a Pandora’s box of unplanned and unanticipated risks^6,8^, as identified here. Our study suggests that majority of the planned and ongoing road-infrastructure expansion projects in Kalimantan are based on unrealistic assumptions about their relative costs and benefits when the full range of environmental, economic, financial, and socio-political factors are considered.

## Conclusion

The currently planned and ongoing expansion of roads and rail lines in Indonesian Borneo will have severe deleterious impacts on native forests in the region. These projects will promote and shape future investments, particularly for logging, mining, and oil palm developments^26^, but will have major impacts on existing forests and wildlife, and will carry serious and poorly recognized economic, financial, social, and political risks. There is an urgent need to reframe the current approach of these projects and infrastructure expansion in Kalimantan as a whole — using a transparent framework that considers the full range of their likely costs and benefits.

## Methods

### Planned and ongoing infrastructure expansion

We obtained spatial data and maps of planned and ongoing roads and rail lines in Kalimantan from a variety of regional and national sources in an endeavour to include the majority of the planned and ongoing roads and rail lines in our analyses. We digitized parallel border roads and other planned and ongoing road developments from infrastructure maps of each of the five provinces of Kalimantan (West Kalimantan, East Kalimantan, Central Kalimantan, South Kalimantan and North Kalimantan)^31–35^ generated from the Indonesian Government Infrastructure Information website (http://loketpeta.pu.go.id/peta-infrastruktur). These maps contain planned and ongoing roads in each province of Kalimantan. We also digitized planned new and upgrade roads of Trans Kalimantan Highways from Potter^38^, and the Indonesian Master Plan (2011–2025)^26^, which identified the planned routes and other planned highways in Kalimantan. Furthermore, we digitized additional planned and ongoing roads in North Kalimantan and West Kalimantan^71^. The digitization procedure entailed geo-referencing maps and then tracing them in a GIS. Subsquently, we retraced the digitized GIS layer in Google Earth to acquire the maximum accuracy with respect to the alignment of the existing roads. The estimated locational error of the final digitized features were <200 m relative to Google Earth. We also compiled spatial data on the planned roads, rail lines and rail stations in North Kalimantan, and on the Samarinda Freeway from the Department of Forestry, Mulawarman University, Samarinda, East Kalimantan (Fig. 1). Extensive discussions to appreciate the ambitions, constraints, and precautions surrounding the planned and ongoing road expansion projects were held with relevant provincial and district planning offices (Bappeda), environmental agencies, Non-Government Organizations (NGOs) such as World Wide Fund for Nature (WWF), and local community groups such as Balikpapan Bay Community Group in Balikpapan. The aim of these discussion was understanding of local pressing issues relevant to these infrastructure expansion projects.

### Forest spatial pattern, and landscape connectivity

We conducted a morphological spatial pattern analysis (MSPA)^43,72^ to describe the spatial pattern of the current and future Kalimantan forests due to the infrastructure expansion. Analyses were performed using Graphical User Interface for the Description of Image Objects and their Shapes (Guidos Toolbox 2.6 version 4)^43,72^. The MSPA described the spatial configurations of Kalimantan forests by segmenting them into distinct elements of the forest landscape, namely core forest patches (forest ≥900 m from the nearest forest edge), edge forest (forest <900 m from an edge), bridge forest - forest corridors connecting different core forest patches, loop forest- forests connecting different sections of a core-forest patch, islet forest- isolated forest patches that are too small to contain core area (details MSPA analysis technique is available in Vogt^43^ and Vogt and Riitters^72^). MSPA thus simultaneously captures changes to both fragmentation and connectivity across a forest landscape while retaining spatially-explicit focus on critical individual forest patches^72^, unlike similar approaches to fragmentation analysis. Our 900-m threshold defining edge forests is taken as a mid-range indicator of potential edge effects on forest patches. Edge effects can extend from a few meters to more than 2000 m from the forest periphery to its interior^46^. An edge effect threshold of 1000 m has been widely used in road impacts research^73^.

To understand landscape connectivity in Kalimantan forest and the potential changes due to the infrastructure expansion we conducted a network component analysis based on the MSPA outputs, using Guidos Toolbox 2.6 version 4^43,72^. The analyses considered forested areas to be internally connected where they were contiguously bridged by at least one 150-m forest pixel. In this way we identified discrete, disconnected forest patches across Kalimantan, consisting of core forests and their links (i.e., bridge forest), and estimated the Equivalent Connected Area (ECA) relative index^43,72^. This index indicates the percentage of the forested areas that are accessible to wildlife population, based on the degree of forest connectivity across the landscape^43,72^.

For the above analyses, we classified primary forests, selectively logged forests, and regrowth forests as “forest class”, without discrimination among these constituent classes. This reflected our objective of evaluating forest connectivity with respect to non-forested areas in the landscape. Here, primary forests are relatively pristine forest without clear human disturbances according to satellite imagery. Selectively logged forests are those that have been disturbed by selective, usuually industrial-scale logging to varying degrees since 1973. Regrowth forests are those that are roughly resembles old-growth forest in canopy structure but were likely young regrowth in 1973 according to satellite imagery^74^. These forest land cover classes were according to latest 30-m Landsat derived land-cover classifications of Gaveau, *et al*.^74^. In this method, a general ‘forest’ class was classified using Landsat satellite imagery for various years between 1973 and 2015, and logging roads were visually interpreted and digitised within the forests of each year of this time series^22,74^. Forest cover within 700 m of contemporary logging roads was then reclassified as ‘logged forest’ given that depressed canopy cover indicative of logging was notable up to 700-m distance as observed by Gaveau, *et al*.^22^. The forest cover datasets were resampled to 150 m spatial resolution for consistency with aforementioned Guidos analyses. We conducted the above analyses for the current landscape and also for the future landscape under projected infrastructure expansion, incorporating a 1 km buffer on both sides of planned and ongoing roads and rail lines (new and upgrade).

### Protected areas, industrial concessions, and peatlands

To spatially evaluate the planned and ongoing infrastructure expansion routes with respect to conservation areas and industrial development, we spatially overlaid planned and ongoing routes over protected areas, peatlands, different forests classes, and industrial concession maps for logging, wood fibre plantation and oil palm plantation in Kalimantan. The protected areas data were obtained from Salim^75^ because these data are more robust at a regional scale than other freely available protected-area datasets such as The World Database on Protected Areas^76^. The protected areas encompass various forest designation, including protection forests, national parks, wildlife reserves, citizen forestry parks, sanctuary reserve, nature recreation parks and nature reserves. The spatial delineation and designation of the protected areas are according to national and provincial spatial plans^75^. The peatlands distribution data were obtained from Wetland International Wetland Program^77^, which was widely used by the Indonesian government, and the different industrial concessions were obtained from Global Forests Watch^78–80^.

### Environmental risks

We categorised planned and ongoing road and rail lines construction and upgrade into four categories to explore potential environmental risks from the respective road segments. Road and rail lines can be environmentally risky, if all road risks are not considered by the road proponents^6,8^. The environmental risks considered in our categorisation are positively linked with habitat importance for wildlife (such as protected area), habitat sensitivity to disturbances (such as peat land), and forest intactness (such as primary forest)^4,11,69,81^. Considering all those notions, and providing 1 km buffer, we categorised planned and ongoing road and rail lines construction and upgrade into four groups: (i) *very high impact* – road and rail line segments that will bisect protected areas; (ii) *high impact –* road and rail line segments that will cut through primary forest or peat land; (iii) *moderate impact* – road and rail line segments that will cut through selectively logged forests or regrowth forests; and (iv) *low impact* – planned and upgrade road and rail line segments that will be located in currently non-forested areas.

## Data Availability

Data available upon request.

## References

[1] Dulac, J. Global land transport infrastructure requirements: Estimating road and railway infrastructure capacity and costs to 2050. 50 (International Energy Agency, Paris, France, 2013).

[2] Laurance, W. F. Conservation and the global infrastructure tsunami: Disclose, debate, delay! *Trends in Ecology & Evolution*, 10.1016/j.tree.2018.05.007 (2018). 10.1016/j.tree.2018.05.007 29910182

[3] Laurance WF, Burgués-Arrea I (2017). Roads to riches or ruin?. Science.

[4] Sloan S (2018). Infrastructure development and contested forest governance threatened the Leuser Ecosystem, Indonesia. Land Use Policy.

[5] Laurance WF, Sloan S, Weng L, Sayer JA (2015). Estimating the environmental costs of Africa’s massive “Development Corridors”. Current Biology.

[6] Alamgir M (2017). Economic, socio-political and environmental risks of road development in the tropics. Current Biology.

[7] Alamgir, M., Campbell, M. J., Sloan, S., Phin, W. E. & Laurance, W. F. In *Jurutera* Vol. February 2018 13–16 (The Institution of Engineers, Malaysia, Malaysia, 2018).

[8] Laurance WF, Goosem M, Laurance SGW (2009). Impacts of roads and linear clearings on tropical forests. Trends in Ecology & Evolution.

[9] Hughes AC (2018). Have Indo-Malaysian forests reached the end of the road?. Biological Conservation.

[10] Sloan, S., Supriatna, J., Campbell, M. J., Alamgir, M. & Laurance, W. F. Newly discovered orangutan species requires urgent habitat protection. *Current Biology*, 10.1016/j.cub.2018.04.082 (2018). 10.1016/j.cub.2018.04.082 29731303

[11] Barber CP, Cochrane MA, Souza CM, Laurance WF (2014). Roads, deforestation, and the mitigating effect of protected areas in the Amazon. Biological Conservation.

[12] Hansen MC (2013). High-resolution global maps of 21st-century forest cover change. Science.

[13] MOEF. Statistics of Environment and Foresty 2016. (Indonesia Ministry of Environment and Forestry, Jakarta, 2016).

[14] Meijaard E, Nijman V (2003). Primate hotspots on Borneo: Predictive value for general biodiversity and the effects of taxonomy. Conservation Biology.

[15] Labrière N, Laumonier Y, Locatelli B, Vieilledent G, Comptour M (2015). Ecosystem services and biodiversity in a rapidly transforming landscape in Northern Borneo. Plos One.

[16] Wich SA (2008). Distribution and conservation status of the orang-utan (Pongo spp.) on Borneo and Sumatra: how many remain?. Oryx.

[17] Posa MRC, Wijedasa LS, Corlett RT (2011). Biodiversity and conservation of tropical peat swamp forests. BioScience.

[18] Myers N, Mittermeier RA, Mittermeier CG, da Fonseca GAB, Kent J (2000). Biodiversity hotspots for conservation priorities. Nature.

[19] Fuller DO, Jessup TC, Salim A (2004). Loss of forest cover in Kalimantan, Indonesia, Since the 1997–1998 El Niño. Conservation Biology.

[20] Curran LM (2004). Lowland forest loss in protected areas of Indonesian Borneo. Science.

[21] Margono BA, Potapov PV, Turubanova S, Stolle F, Hansen MC (2014). Primary forest cover loss in Indonesia over 2000–2012. Nature Clim. Change.

[22] Gaveau DLA (2014). Four decades of forest persistence, clearance and logging on Borneo. Plos One.

[23] Gaveau DLA (2016). Rapid conversions and avoided deforestation: examining four decades of industrial plantation expansion in Borneo. Scientific Reports.

[24] Abood SA, Lee JSH, Burivalova Z, Garcia-Ulloa J, Koh LP (2015). Relative contributions of the logging, fiber, oil palm, and mining industries to forest loss in Indonesia. Conservation Letters.

[25] BPS. Statistics Indonesia. (Ministry of Public Works and Provincial/Regency Public Works Offices, Jakarta, 2017).

[26] Coordinating Ministry For Economic Affairs. Masterplan for Acceleration and Expansion of Indonesia Economic Development 2011–2025. 205 (Coordinating Ministry For Economic Affairs, Republic of Indonesia, Jakarta, 2011).

[27] Sloan, S. *et al*. Hidden challenges for conservation and development along the Papuan economic corridor. *Environmental Science and Policy* **92**, 98–106, 10.1016/j.envsci.2018.11.011 (2019).

[28] Indonesia Infrastructure Initiative. Vol. 18 March 2016 (2016).

[29] Moerwanto, A. S. In *107*^*th*^*Meeting of REAAA Governing Council* 30 (Mania, 2017).

[30] The Jakarta Post. Vol. March 12, 2016 (Jakarta, Indonesia, 2016).

[31] Indonesia Government. Infrastructure map of West Kalimantan province. (Ministry for Public Works and Human Settlement, Jakarta, 2016).

[32] Indonesia Government. Infrastructure map of South Kalimantan province. (Ministry for Public Works and Human Settlement, Jakarta, 2016).

[33] Indonesia Government. Infrastructure map of East Kalimantan province. (Ministry for Public Works and Human Settlement, Jakarta, 2016).

[34] Indonesia Government. Infrastructure map of Central Kalimantan province. (Ministry for Public Works and Human Settlement, Jakarta, 2016).

[35] Indonesia Government. Infrastructure map of North Kalimantan province. (Ministry for Public Works and Human Settlement, Jakarta, 2016).

[36] Handayani, O. In *Indonesia Expat* Vol. May 22, 2017 (Jakarta, Indonesia, 2017).

[37] Winosa, Y. In *JakartaGlobe* Vol. July 14, 2016 (Jakarta, Indonesia, 2016).

[38] Potter L (2009). Resource periphery, corridor, heartland: Contesting land use in the Kalimantan/Malaysia borderlands. Asia Pacific Viewpoint.

[39] Strategic Asia. Implementing Indonesia’s Economic Master Plan (MP3EI): challenges, limitations and corridor specific differences. 87 (Strategic Asia, 2012).

[40] Wich SA (2012). Understanding the Impacts of Land-Use Policies on a Threatened Species: Is There a Future for the Bornean Orang-utan?. Plos One.

[41] Laurance, W. F. In imperiled forests of Borneo, a rich tropical eden endures. *Yale Environment 360* (2013).

[42] Dounias, E. Borneo’s bearded pig, gardener of forests and protector of their inhabitants. *The Conversation* (2018).

[43] Vogt, P. User guide of Guidos Toolbox. 43 (European Commission Joint Research Centre (JRC), Italy, 2017).

[44] Laurance SGW, Gomez MS (2005). Clearing width and movements of understory rainforest birds. Biotropica.

[45] Watson, J. E. M. *et al*. The exceptional value of intact forest ecosystems. *Nature Ecology & Evolution*, 10.1038/s41559-018-0490-x (2018). 10.1038/s41559-018-0490-x 29483681

[46] Laurance WF (2002). Ecosystem decay of Amazonian forest fragments: a 22-year investigation. Conservation Biology.

[47] Campbell MJ (2017). Forest edge disturbance increases rattan abundance in tropical rain forest fragments. Scientific Reports.

[48] Campbell, M. J. *et al*. Edge disturbance drives liana abundance increase and alteration of liana–host tree interactions in tropical forest fragments. *Ecology and Evolution*, 10.1002/ece3.3959 (2018). 10.1002/ece3.3959 PMC5916267 29721294

[49] Laurance WF (2002). Predictors of deforestation in the Brazilian Amazon. Journal of Biogeography.

[50] Bryan JE (2013). Extreme differences in forest degradation in Borneo: comparing practices in Sarawak, Sabah, and Brunei. PLOS ONE.

[51] Laurance WF (2016). Lessons from research for sustainable development and conservation in Borneo. Forests.

[52] Maisels F (2013). Devastating decline of forest elephants in Central Africa. Plos One.

[53] Blake S (2008). Roadless wilderness area determines forest elephant movements in the Congo Basin. Plos One.

[54] Blake S (2007). Forest elephant crisis in the Congo Basin. Plos Biology.

[55] Clements GR (2014). Where and how are roads endangering mammals in Southeast Asia’s forests?. Plos One.

[56] Wich, S., Riswan, Jenson, J., Refisch, J. & Nellemann, C. Orangutans and the economics of sustainable forest management in Sumatra. 84 (GRASP/PanEco/YEL/ICRAF/GRID-Arendal, 2011).

[57] Laurance WF (2008). Impacts of roads, hunting, and habitat alteration on nocturnal mammals in African rainforests. Conservation Biology.

[58] Laurance WF (2006). Impacts of roads and hunting on Central African rainforest mammals. Conservation Biology.

[59] Clements R (2010). Trio under threat: can we secure the future of rhinos, elephants and tigers in Malaysia?. Biodiversity and Conservation.

[60] CBD. Decision UNEP/CBD/COP/DEC/X/2 Adopted by the Conference of the Parties to the Convention on Biological Diversity at Its Tenth Meeting. (Convention on Biological Diversity (CBD), 2010).

[61] Saura S, Bastin L, Battistella L, Mandrici A, Dubois G (2017). Protected areas in the world’s ecoregions: How well connected are they?. Ecological Indicators.

[62] Saura S (2018). Protected area connectivity: Shortfalls in global targets and country-level priorities. Biological Conservation.

[63] Brun C (2015). Analysis of deforestation and protected area effectiveness in Indonesia: A comparison of Bayesian spatial models. Global Environmental Change.

[64] Laurance WF (2012). Averting biodiversity collapse in tropical forest protected areas. Nature.

[65] Page SE, Rieley JO, Banks CJ (2011). Global and regional importance of the tropical peatland carbon pool. Global Change Biology.

[66] Miettinen J, Shi C, Liew SC (2016). Land cover distribution in the peatlands of Peninsular Malaysia, Sumatra and Borneo in 2015 with changes since 1990. Global Ecology and Conservation.

[67] Laurance WF (2015). Reducing the global environmental impacts of rapid infrastructure expansion. Current Biology.

[68] Laurance WF, Sayer J, Cassman KG (2014). Agricultural expansion and its impacts on tropical nature. Trends in Ecology & Evolution.

[69] Laurance WF (2014). A global strategy for road building. Nature.

[70] Laurance WF, Balmford A (2013). A global map for road building. Nature.

[71] ADB. INO: Regional roads development project. 60 (Asian Development Bank, 2011).

[72] Vogt P, Riitters K (2017). GuidosToolbox: universal digital image object analysis. European Journal of Remote Sensing.

[73] Sloan S, Jenkins CN, Joppa LN, Gaveau DLA, Laurance WF (2014). Remaining natural vegetation in the global biodiversity hotspots. Biological Conservation.

[74] Gaveau, D. L. A., Salim, M. & Arjasakusuma, S. Deforestation and industrial plantations development in Borneo. (Center for International Forestry Research (CIFOR) Data verse, 2016).

[75] Salim, M. Protected area of Borneo. (CIFOR, Bogor, Indonesia, 2015).

[76] IUCN & UNEP-WCMC. The World Database on Protected Areas. (UNEP-WCMC, Cambridge, UK, 2015).

[77] Wahyunto, S. & Subagjo, S. R. H. Maps of area of peatland distribution, area and carbon content in Kalimantan, 2000–2002. 1st edn. Book 1. (Wetlands International - Indonesia Programme & Wildlife Habitat Canada, Bogor, Indonesia, 2004).

[78] GFW. Oil Palm Concessions, Indonesia. (Global Forest Watch, 2018).

[79] GFW. Wood Fibre Concessions, Indonesia. (Global Forest Watch, 2018).

[80] GFW. Logging Concessions, Indonesia. (Global Forest Watch, 2018).

[81] Koh LP, Miettinen J, Liew SC, Ghazoul J (2011). Remotely sensed evidence of tropical peatland conversion to oil palm. Proceedings of the National Academy of Sciences.

